# Adolescents’ and Young Adults’ Perceptions of a Pop-Up Aimed at Combating the Spread of E-Cigarette Misinformation on Social Media: Cross-Sectional Survey Study

**DOI:** 10.2196/73193

**Published:** 2025-08-19

**Authors:** Jessica Maturo, Shivani Mathur Gaiha

**Affiliations:** 1 Division of Adolescent/Young Adult Medicine, Department of Pediatrics, Boston Children's Hospital, 1 Autumn St, 5th Fl, Boston, MA, 02115, United States, 1 6173553538; 2 Boston University School of Public Health, Boston, MA, United States; 3 Department of Pediatrics, Harvard Medical School, Boston, MA, United States

**Keywords:** electronic nicotine delivery systems, vaping, adolescents and young adults, social media, misinformation, prevention, intervention, acceptability, tobacco control

## Abstract

**Background:**

Social media is an important source of e-cigarette–related information for adolescents and young adults. However, misinformation is being shared across platforms, which may encourage e-cigarette use.

**Objective:**

This study aims to examine adolescent and young adult perceptions of a novel pop-up on social media that provides links to vaping–related health information from credible sources (eg, CDC).

**Methods:**

Between December 2023 and March 2024, participants aged 13‐24 years (N=5326) completed a web-based cross-sectional survey. Participants were asked to select from 4 positive (eg, useful to share, helpful to check health-related information) and 4 negative or neutral sentiments (eg, usually ignore such notifications, would not trust it) to reflect their perceptions about a mock pop-up that provided a link to e-cigarette–related information on social media.

**Results:**

More participants endorsed positive sentiments than negative or neutral sentiments in relation to the pop-up. Specifically, 1078 (20.8%) endorsed two or more positive sentiments, and 690 (13.3%) endorsed two or more negative or neutral sentiments when searching for “vaping” on social media; similarly 913 (17.6%) participants endorsed two or more positive sentiments and 690 (13.3%) endorsed two or more negative or neutral sentiments when viewing e-cigarette–related posts in their feed (all *P*<.001). Among those who were searching for e-cigarette–related information, participants aged 13-18 years were more likely to endorse at least two positive sentiments compared to those aged 19‐24 years (ie, 646, 22.0% vs 423, 19.2%, respectively), those who had never used e-cigarettes compared with those who had ever used them (ie, 674, 23.6% vs 404, 17.3%, respectively), and those who last used e-cigarettes more than 30 days ago compared with those who had used them in the past 30 days (ie, 187, 19.8% vs 217, 15.6%, respectively). Similarly, among participants who viewed e-cigarette–related posts in their feed, those who had never used e-cigarettes were more likely to endorse two or more positive perceptions compared to those who had ever used e-cigarettes (ie, 563, 19.7% vs 350, 15.0%), and those who had last used e-cigarettes more than 30 days ago (ie, 159, 16.9% vs 191, 13.7%) were more likely to endorse two or more positive perceptions compared to those who had used them in the past 30 days (all *Ps*<.001). Participants who had never used e-cigarettes were also less likely to trust pop-ups, compared to those who had ever used them, whether while searching for e-cigarette-related information on social media or while seeing e-cigarette-related posts in their feed (approximately, 19%vs 24%). There were no significant differences in the endorsement of negative or neutral sentiments. However, participants aged 13‐18 years were more likely to ignore such notifications while viewing e-cigarette–related posts in their social media feed compared to those aged 19‐24 years (ie, 850, 28.9% vs 563 25%); further, participants who had never used e-cigarettes were less trusting of e-cigarette–related information while searching for vaping or in their feed.

**Conclusions:**

Positive perception of a social media pop-up indicates its potential to prevent e-cigarette–related misinformation. Further development of a pop-up requires strategies to better engage and inform adolescents and young adults, specifically younger individuals, as they may be more likely to disregard pop-ups, and older individuals and those who used e-cigarettes in the past 30 days, as they are potentially more dismissive of such information.

## Introduction

E-cigarette–related misinformation is widely shared on social media, which can lower perceived health risks and undermine adolescents’ and young adults’ quit attempts [1]. As extensive social media use is associated with e-cigarette use among adolescents and young adults [2-4], and seeking information about e-cigarettes predicts future use [5], it is crucial that the information being accessed is accurate and up to date.

Currently, social media platforms combat e-cigarette–related misinformation by restricting branded content by influencers, requiring nicotine health warnings on advertisements [6] and removing reported content [7]. During the COVID-19 pandemic, Instagram implemented graphic pop-ups that directed users to websites of health authorities and other evidence-based resources, which helped connect people with trustworthy information [8]. To date, only one study assessed young adults’ perceptions of links to e-cigarette cessation resources embedded within fictitious provaping influencer posts. Participants who viewed the link had lower odds of susceptibility to e-cigarette use, and higher odds of believing that the product would not cause positive experiences and would cause negative experiences, with no impact on intention to use or desire to quit using e-cigarettes [9]. This study expands upon research to inform social media users about e-cigarette use, by examining adolescent and young adult perceptions of the utility and acceptability of a pop-up that appears both while searching for e-cigarettes on social media and below e-cigarette–related posts, providing a link to information about the harms of tobacco and vaping from their social media feeds.

## Methods

### Participant Recruitment

Between December 2023 and March 2024, the study team administered a national, cross-sectional survey to an online panel of participants aged 13-24 years through Qualtrics. . Recruitment initially aimed for a 3:2 ratio of participants aged 13‐17 and 18‐24-years; however, this restriction was lifted on February 9, 2024, due to low response rates among younger participants. Sampling quotas were used to align sex and race/ethnicity distributions with the latest US Census data. Of 27,525 individuals who accessed the survey, 12,919 were ineligible, 8,064 were excluded for providing incomplete responses, and 1216 were excluded for failing quality checks (eg, completing the survey in under half the median time).

### Sample

The participants (N=5326) had a mean age of 18.4 (SD 3.2) years; 3013 (56.6%) identified as female, and 3207 (60.2%) were non-Hispanic White. Approximately one-quarter (1418; 26.6%) of participants had used an e-cigarette in the past 30 days, 970 (18.2%) had used an e-cigarette but not in the past 30 days, and 2938 (55.2%) had never used an e-cigarette.

### Ethical Considerations

The study was approved by Stanford University’s School of Medicine IRB (Protocol Number IRB-54761). We collected e-consent from participants 18 years and above and assent from participants under 18 years for this anonymous survey (with a waiver of documentation of parental consent). Each participant could complete the survey only once and received up to US $5 or a panel-specific incentive.

### Measures

E-cigarette use status was measured by asking participants whether they had ever used a nicotine e-cigarette, even one or two puffs. Response options included “yes, in the past 30 days,” “yes, but not in the past 30 days,” and “no.”

**Figure 1.**
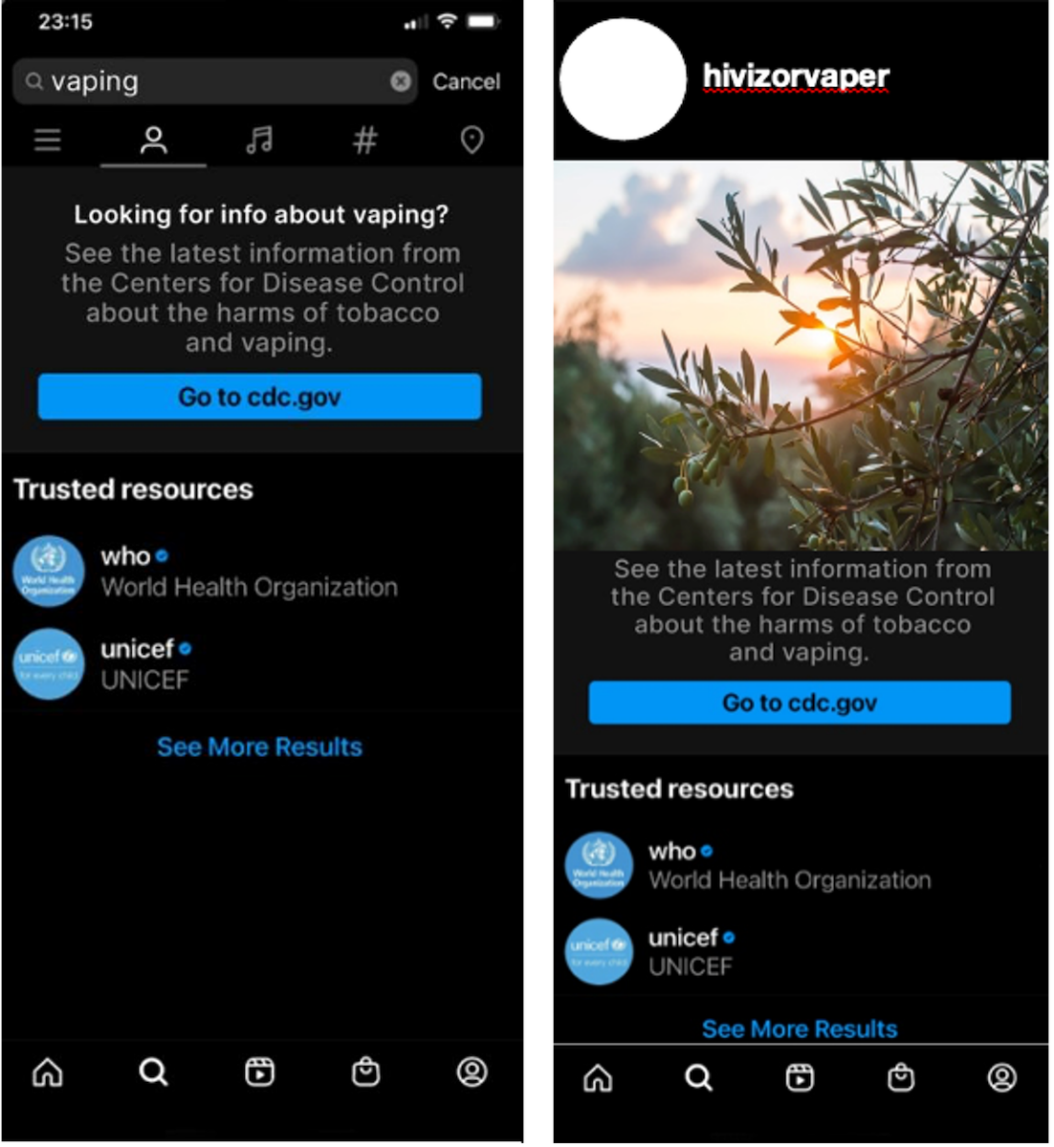
Two images of fictitious social media pop-ups appearing when searching for information about “vaping” and when viewing posts in one’s social media feed.

Participants were shown two images of a fictitious pop-up tagged as “trusted resources” with a link to “vaping–related information” from the Centers for Disease Control and Prevention (CDC), World Health Organization (WHO), and United Nations Children’s Fund (UNICEF) (Figure 1). They were then asked what they would think about seeing this pop-up appear when: (1) searching for vaping on social media (“Imagine you were searching for vaping on social media and a link came up like in the image shown below. What would you think?”) and (2) looking at social media posts in their feed (“Imagine you were looking at social media posts and a link came up like in the image shown below. What would you think?”). Eight response options exhibiting 4 positive and 4 negative or neutral perceptions were provided; participants could select all that applied (see list of all perceptions in tabulated results). Additionally, participants had the option to select ‘other’ and write-in their own response. We also asked participants where they would prefer a link or trusted resource to appear.

### Analysis

Descriptive analyses examined the proportion of participants endorsing individual perceptions as well as two or more positive and negative or neutral sentiments. Chi-square analyses were used to examine differences in the proportion of participants endorsing individual perceptions as well as two or more positive and negative or neutral perceptions across age groups (13‐18 y vs 19‐24 y) and by e-cigarette use status (those who have never used vs those who have used; those who have used in the past 30 d vs those who used but not in the past 30 d). A significance level of *P*<.05 was used to determine if the proportions differed statistically between groups (ie, one group was more or less likely to endorse a particular perception than the other).

## Results

### Perceptions of a Social Media Pop-Up

Among participants who viewed a pop-up intended to appear while searching for information on e-cigarettes (n=5186), positive sentiments endorsed were: the pop-up was good for checking health-related information (24.9%), might help prevent misinformation (24.2%), provided useful information about e-cigarettes that is a click away (23.2%), and would help to share information with peers (15.1%) (Table 1). Negative or neutral sentiments endorsed included: usually ignoring such notifications (27.3%), not trusting the pop-up (22.2%), perceiving that it would not help with the real problem that lies with sellers online or on social media (16.6%), and believed that it was for people who did not really know anything about vaping (15.7%). When asked about searching for information, 20.8% endorsed two or more positive sentiments and 13.3% endorsed two or more negative or neutral sentiments (*P*<.001).

**Table 1.** Participants endorsing positive and negative perceptions towards a pop-up, stratified by age and e-cigarette use status.

Endorsing perceptions towards the pop-up	Participants (N=5326), n (%)	Age (N=5326), n (%)		E-Cigarette use status (N=5326), n (%)		E-cigarette users (n=2388), n (%)	
		13‐18 years (n =3037)	19‐24 years (n=2289)	Never used (n=2938)	Ever used (n=2388)	Used >30 days ago (n=970)	Past 30-day use (n=1418)
While searching for “vaping” on social media (n=5186)							
Two or more positive perceptions	1078 (20.8)	646 (22.0)^a^	432 (19.2)	674 (23.6)^a^	404 (17.3)	187 (19.8)^a^	217 (15.6)
Two or more negative or neutral perceptions	690 (13.3)	391 (13.3)	299 (13.3)	375 (13.2)	315 (13.5)	112 (11.9)	203 (14.6)
Positive perceptions							
Good to check health-related information about vaping	1293 (24.9)	755 (25.7)	538 (24.0)	784 (27.5)^a^	509 (21.8)	203 (21.5)	306 (22.0)
May help prevent misinformation	1254 (24.2)	734 (25.0)	520 (23.2)	741 (26.0)^a^	513 (22.0)	236 (25.0)^a^	277 (19.9)
Useful information about vaping that is a click away	1203 (23.2)	719 (24.5)^a^	484 (21.6)	697 (24.5)^a^	506 (21.7)	217 (23.0)	289 (20.8)
Will help to share information with friends and others my age	785 (15.1)	448 (15.2)	337 (15.0)	435 (15.3)	350 (15.0)	148 (15.7)	202 (14.5)
Negative/neutral perceptions							
I usually ignore these notifications	1417 (27.3)	828 (28.2)	589 (26.2)	729 (25.6)^a^	688 (29.5)	259 (27.4)	429 (30.8)
I don’t think I will trust it	1153 (22.2)	657 (22.3)	496 (22.1)	700 (24.4)^a^	453 (19.4)	198 (21.0)	255 (18.3)
It won’t help with the real problem which is people who sell online or on social media	861 (16.6)	492 (16.7)	369 (16.4)	462 (16.2)	399 (17.1)	165 (17.5)	234 (16.8)
I think that’s for people who really don’t know anything about vaping	812 (15.7)	420 (14.3)^a^	392 (17.5)	420 (14.7)^a^	392 (16.8)	149 (15.78)	243 (17.5)
While looking at social media posts in their feed (n=5189)							
Two or more positive	913 (17.6)	533 (18.1)	380 (16.9)	563 (19.7)^a^	350 (15.0)	159 (16.9)^a^	191 (13.7)
Two or more negative/neutral	690 (13.3)	395 (13.4)	295 (13.1)	370 (13.0)	320 (13.7)	117 (12.5)	203 (14.6)
Positive perceptions							
Good to check health-related information about vaping	1134 (21.9)	650 (22.1)	484 (21.5)	663 (23.2)^a^	471 (20.2)	192 (20.5)	279 (20.0)
May help prevent misinformation	1105 (21.3)	661 (22.5)	444 (19.8)	654 (22.9)^a^	451 (19.3)	194 (20.7)	257 (18.4)
Useful information about vaping that is a click away	1101 (21.2)	612 (20.8)	489 (21.8)	617 (21.6)	484 (20.7)	185 (19.7)	299 (21.4)
Will help to share information with friends and others my age	763 (14.7)	428 (14.6)	335 (14.9)	433 (15.2)	330 (14.1)	130 (13.8)	200 (14.3)
Negative/neutral perceptions							
I usually ignore these notifications	1413 (27.2)	850 (28.9)^a^	563 (25.0)	767 (26.9)	646 (27.7)	251 (26.7)	395 (28.3)
I don’t think I will trust it	1144 (22.1)	669 (22.8)	475 (21.1)	695 (24.3)^a^	449 (19.2)	202 (21.5)^a^	247 (17.7)
It won’t help with the real problem which is people who sell online or on social media	862 (16.6)	472 (16.1)	390 (17.4)	456 (16.0)	406 (17.4)	159 (16.9)	247 (17.7)
I think that’s for people who really don’t know anything about vaping	873 (16.8)	462 (15.7)^a^	411 (18.3)	428 (15.0)^a^	445 (19.1)	163 (17.4)	282 (20.2)

a *P* values significant at *P*=.05 level.

b *χ*2 analyses exclude missing data.

c The maximum percentage missing across all groups was 2.63%.

d *χ*2 analyses assessed differences in the proportion of participants endorsing two or more positive versus two or more negative/neutral perceptions. Participants were significantly more likely to endorse two or more positive perceptions than negative/neutral perceptions when searching for vaping information and looking at posts in the feed (both *P*<.0001).

While viewing e-cigarette–related posts, a pop-up intended to appear in their feed (n=5189), positive sentiments endorsed by participants included that the pop-up was good to check health-related information (21.9%), might help prevent misinformation (21.3%), provided useful information about e-cigarettes that was a click away (21.2%), and would help to share information with peers (14.7%). Negative or neutral sentiments endorsed were that they usually ignore such notifications (27.2%); would not trust it (22.1%); it would not help with the real problem, which is sellers online or on social media (16.6%); and that it is for people who did not really know anything about vaping (16.8%). While viewing e-cigarette-related posts, 17.6% endorsed two or more positive sentiments and 13.3% endorsed two or more negative or neutral sentiments (*P*<.001).

### Differences in Perceptions Based on Age Group and E-Cigarette Use Status

While searching for “vaping” on social media, 13‐18-year-old participants were more likely to endorse two or more positive perceptions compared to 19‐24-year old participants (22.0% vs 19.2%, respectively). Furthermore, 13‐18-years old participants were more likely to think the pop-up offered useful information just a click away (24.5% vs 21.6%) and less likely to believe that the pop-up was for people who did not know anything about vaping (14.3% vs 17.5%) compared to 19‐24-year-old participants. Participants who had never used e-cigarettes compared to those who had ever used (23.6% vs 17.3%) and those with e-cigarette use>30 days ago compared to those with past-30-day use (19.8% vs 15.6%) were more likely to endorse two or more positive perceptions while searching for e-cigarette information. Participants who had never used e-cigarettes were significantly more likely to find pop-ups good for checking health-related information about e-cigarettes (27.5% vs 21.8%), helpful in preventing misinformation (26.0% vs 22.0%), and useful in providing information about e-cigarettes that was a click away (24.5% vs 21.7%) compared to those who ever used. Moreover, those who had never used e-cigarettes were more likely to say they would not trust the pop-up (24.4% vs 19.4%) and less likely to say they usually ignored notifications on social media (25.6% vs 29.5%) compared to those who had ever used them. Participants who used e-cigarettes >30 days ago were more likely to say that the pop-up may help prevent misinformation compared to those with past-30-day use (25.0% vs 19.9%, respectively).

While viewing social media posts in their feed, 13‐18-year-old participants were more likely to say they usually ignored such notifications (28.9% vs 25.0%) and were less likely to think that the pop-up was for people who did not know anything about vaping (15.7% vs 18.3%) compared to 19‐24-year-olds. Participants who had never used e-cigarettes compared to those who had used (19.7% vs 15.0%), and those with use >30 days ago compared to those with past-30-day use (16.9% vs 13.7%) were more likely to endorse two or more positive perceptions. Those who had never used e-cigarettes were more likely to say the pop-up was good to check health-related information about vaping (23.2% vs 20.2%) and may prevent misinformation (19.3% vs 22.9%) compared to those who have ever used. In addition, participants who never used e-cigarettes compared to those who had ever used (24.3% vs 19.2%) and those who had used>30 days ago compared to those with past-30-day use (21.5% vs 17.7%) were more likely to say they would not trust the pop-up.

### Location Preference for Link or Trusted Resource

When asked where they would prefer a link or a trusted resource to appear (n=5,136), 25.2% participants indicated below the post but above comments, 20.7% at the top of the post with the post slightly greyed out, 19.6% as the next post, reel, or TikTok, 18.8% as a pop-up, and 15.8% below the post and comments.

## Discussion

Overall, participants viewed the pop-up more positively than negatively or neutrally. The pop-up was viewed more favorably by those who had never used e-cigarettes than those who had used e-cigarettes, by those who used >30 days ago than current users, and by younger participants (under 18 y) compared to those older than them. One explanation is that adolescents and young adults who use e-cigarettes may be less likely to worry about associated health risks and therefore may not perceive the pop-up as beneficial or necessary [10].

Our finding that those who had never used e-cigarettes were less likely to trust the pop-up compared to those who had used, aligns with research demonstrating that noncigarette smokers were less likely to trust health information from online sources compared to current or former cigarette smokers [11]. Future research should assess if adolescents and young adults who have never used e-cigarettes are more likely to seek out information about vaping than those who had used them, which could potentially explain why they held specific positive perceptions.

Furthermore, older participants were less likely to have two or more positive perceptions compared to younger individuals. One explanation is that older adolescents may have more experience using social media, and as a result they may be less likely to click on pop-ups due to the association between pop-up windows and unwanted and inappropriate content, malware, spam advertisements, and high distractibility [12,13]. Another potential reason why older participants were less likely to have positive perceptions of the pop-up is that because older adolescents and young adults (now aged 18‐24 y) were in middle or high school during the peak of the youth e-cigarette epidemic in 2019, which may have likely resulted in increased exposure to e-cigarette-use behavior and normalized use or anti-e-cigarette and related messaging, and that they may have more entrenched beliefs about e-cigarettes. Thus, they may be less responsive to a pop-up providing additional health information about e-cigarettes in the present day.

One limitation of this formative study is that because the pop-up was presented hypothetically, it is unclear how participants would perceive the pop-up in real-time on social media. In addition, although roughly five percent of the participants did not answer the question or wrote-in their own response, it is possible that the list of response options did not exhaustively capture all possible perceptions.

Nonetheless, our study on adolescent and young adult perceptions can inform the potential development of a social media pop-up to prevent misinformation and e-cigarette use. Additional research is needed on how to reach out to adolescents and young adults on social media and strategies to make pop-ups or other notifications more favorable among adolescents and young adults, especially older individuals and those with past 30-day use who appear less receptive to information in such notifications. Further, it may be beneficial to co-design social media outreach with youth to ensure that e-cigarette–related information and notifications are not disregarded by younger individuals on social media. Moreover, examining factors that facilitate or inhibit trust in e-cigarette–related information on social media can provide information and recommendations for social media policy initiatives.

## Abbreviations

CDC: Centers for Disease Control and Prevention
